# Psychosocial Factors Associated With Increased Adolescent Non-suicidal Self-Injury During the COVID-19 Pandemic

**DOI:** 10.3389/fpsyt.2021.743526

**Published:** 2021-12-10

**Authors:** Na Du, Yingjie Ouyang, Yu Xiao, Yunge Li

**Affiliations:** Clinical Psychology Department, The Fourth People's Hospital of Chengdu, Chengdu, China

**Keywords:** adolescent, COVID-19, NSSI, psychosocial factors, during-pandemic

## Abstract

Non-suicidal self-injury (NSSI) in adolescents hospitalized with psychiatric disorders continues to increase after the outbreak of COVID-19. This study aimed to explore the relationship between the pandemic and NSSI among adolescents and whether the composition of psychosocial factors related to NSSI has changed during the pandemic. Through the retrospective analysis of medical records retrieved from the electronic system of the psychiatric hospital located in Chengdu from January 2016 to March 2021, 609 medical records of adolescents were obtained. The main potential psychosocial factors were determined by deductive content analysis. Among the 609 adolescents, 420 subjects had engaged in NSSI, while 189 did not. We found that the percentage of adolescents who had engaged in NSSI in 2016 was only 29.2%, reaching 34.5% in 2017, 45.7% in 2018, 61.3% in 2019, 92.5% in 2020, and 95.9% in 2021. In the binary logistic regression model, female sex (OR = 0.073, 95% CI: 0.028–0.186), older age (OR = 1.234, 95% CI: 1.030–1.478), having a single parent (OR = 7.865, 95% CI: 3.997–15.476), having experienced trauma (OR = 2.192, 95% CI: 1.032–4.654), having experienced social isolation from peers (OR = 8.139, 95% CI: 4.037–16.408), having experienced body-focused bullying (OR = 3.078, 95% CI: 1.295–7.318), overuse of a mobile phone in the parents' opinions (OR = 4.354, 95% CI: 1.380–13.738), having attempted suicide (OR = 9.120, 95% CI: 4.492–18.512), and during the pandemic (time point is January 30, 2020) (OR = 5.399, 95% CI: 1.679–17.357) were the factors that were significantly associated with NSSI. When comparing the differences in psychosocial factors between the pre-pandemic and the during-pandemic groups, the results showed that the family constitution, parent–child relationships, mobile phone overuse, and stressful learning were important factors. Tailored interventions geared towards changed psychosocial factors should be formulated.

## Introduction

NSSI (non-suicidal self-injury) refers to the behaviour of directly damaging one's body tissue, such as self-harming, skin scratching, and self-burning (1). According to the definition, the purpose of implementing this behaviour is not to cause death, but many related studies have found that the suicide risk of patients who engage in NSSI is hundreds of times higher than that of the general population (2, 3). NSSI brings a severe burden to the society and families. Some scholars have found through epidemiological investigation that the incidence rate of NSSI is 14–39% in the general adolescent population, while it is as high as 40–61% in adolescent inpatients (4). Due to its high detection rate, high risk, and high repeatability, this behaviour has become one of the most important public health problems in the world.

In recent years, there have been many studies aimed at exploring the causes of NSSI among adolescents. According to Chapman's Experiential Avoidance Model, individuals engage in NSSI to escape from unwanted emotions, which is primarily maintained by negative reinforcement (5). Casey and colleagues revealed that adolescents are vulnerable to NSSI because of their elevated levels of impulsivity and emotional reactivity (6). Beyond emotional reasons, demographic factors such as female sex (7) were found to be related to NSSI. Other risk factors for NSSI might focus on psychosocial factors, including dysfunctional relationships (8), being bullied by peers (9), and being mistreated by parents (10). Among all these risk factors, it was found that accumulated stressors play an important role in the onset of NSSI (11). Adolescents living in modern society experience many stressors (12), and it was estimated that by 2020, there would be 15–30 million teenagers who engage in NSSI (13). In addition to the increased stressors mentioned above, a sudden COVID-19 pandemic hit us in early 2020, which greatly changed our life, and the changed lifestyle might make people anxious (14). Compared to adults, the particular group of children and adolescents, who constitute ~ 28% of the world's population (15), have more vulnerability factors, which increases the impact of the pandemic on their lives (16). The latest studies have reported that the pandemic has caused adolescents to be unable to meet friends, unable to participate in outdoor activities, and unable to engage in school activities, which may have continuous negative effects on their mental health (17, 18). Undoubtedly, the COVID-19 pandemic is a strong stressor for adolescents, which might further increase their NSSI prevalence, like the experience reported by Hasking et al., (19). It can be demonstrated by the findings in the research by Ougrin et al., that the pandemic-related emergency psychiatric presentations of NSSI for children and adolescents increased from 50% in 2019 to 57% during the COVID-19 lockdown in 2020 (20).

Although the psychosocial factors for NSSI have been studied in depth, there is little literature that points out the composition and structural changes of these factors brought about by the COVID-19 pandemic, and it remains to be determined what reasons have caused the increase in NSSI among hospitalized adolescents in psychiatric hospitals. To answer these questions, we performed a retrospective analysis of the medical record data of adolescent patients treated in the Clinical Psychology Department of the Fourth People's Hospital of Chengdu from 2016 to present. The purpose of our study was 1) to determine if the pandemic is associated with an increase in NSSI among adolescents; 2) to explore whether the composition of related psychosocial factors related to NSSI has changed during the pandemic; and 3) to provide tailored measures for the prevention of NSSI in the future.

## Materials and Methods

### Participants

Our study was a retrospective analysis of medical record data retrieved from the electronic medical record system of the Fourth People's Hospital of Chengdu. This hospital is responsible for the treatment of all types of common psychiatric disorders, and the patients were mainly from major cities in Sichuan Province, sometimes from cities outside the province. It could be said that the population coverage rate of this hospital is still relatively large. The key limitations for retrieval were the patients' age ≤ 18 years, who were admitted to the Clinical Psychology Department of the Fourth People's Hospital of Chengdu during the period from January 2016 to March 2021. It is important to note that from February 2020 to March 2020, this ward was shut down due to the pandemic, so the number of cases in these 2 months was 0. (This ward was mainly responsible for treating patients with anxiety, depression, and other mood disorders.) Irrelevant data were filtered out, including the data of adults over 18 years old and data from repeat hospitalizations; for the latter, we only chose the latest medical record for analysis. In the initial records search, the number of patients hospitalized repeatedly was 23. A total of 609 medical records were obtained, all of which were approved for use by the patients' parents at the time of admission.

### Measures

Four members in this study read through the medical records of each case, and according to their contents of the records, they included the information of the patients' biological sex, age, duration of disorder, the frequency of treatment episodes (it referred to the times of treatment for their psychiatric disorders before being admitted to our hospital, including the medication and psychotherapy), psychiatric diagnoses (The diagnoses were made according to the International Classification of Diseases, ICD, the 10th version, by the diagnosis and treatment team in the ward through the professional rounds. The team consisted of three psychiatrists who had worked in this field for at least 10 years.), the date of hospitalization, the marital status of their parents, whether they were only children, and their lifetime history of NSSI behaviours and suicide attempts. All these data were recorded into the Excel spreadsheet created on the computer. NSSI was defined as any act of self-injury associated with no intent to die, including the intentional self-cutting, self-burning, self-biting, self-scratching, and self-punching. NSSI was assessed during admission *via* a psychiatric interview conducted by attending physicians. The psychiatric interview was implemented in a standardized process, which could gain the information of the patients' upbringing history, including their relationship with peers and parents. In this evaluation, all patients were asked whether they had ever engaged in NSSI. If they said yes, physicians needed to record the way, the frequency, and the time of the latest self-injury behaviour into the medical records. Simultaneously, physicians were responsible for checking the scars or damaged body tissue caused by NSSI during the physical examination session. To determine whether the pandemic was associated with NSSI, we also added an item named “Before/During the pandemic” to classify the data. According to the statement by the World Health Organization, which declared January 30, 2020, as the start date of the outbreak of COVID-19 (21), we defined this date as the time point; namely, the patients admitted before this time were considered as “before the pandemic.” The spreadsheet also includes items related to psychosocial factors of NSSI behaviours, which were selected by the members in this study by deductive and inductive content analysis. (The specific process was described in section Statistical analysis) The research received institutional review board (IRB) approval from the Fourth People's Hospital of Chengdu.

### Statistical Analysis

The study combined qualitative and quantitative analyses. For the qualitative part, deductive content analysis was utilized to identify the main related psychosocial factors of NSSI. On the basis of a literature review, we developed an unconstrained classification matrix, which focused on the relevant factors of NSSI. The identified categories, including “Experienced trauma,” “Body-focused bullying,” “Social isolation from peers,” “Hurt by information on social media,” and “Parent–child relationships,” were all retrieved from previously published studies (22–28). Then, four members in this study read all 609 medical records and coded the data for correspondence with the five identified categories. Under the item “Parent–child relationships,” we found that most of the records had a similar phrase: “parents didn't understand my feelings, and they often misunderstood me.” Therefore, we changed the title to “Parents' misunderstanding” and used it in Table 1. During our work, we found some factors that did not fit the categorization frame, and then we followed the principles of inductive content analysis to create the new categories (after discussing collectively, until consensus was reached, the lists of headings were grouped under higher-order subthemes. As a result, the subthemes of “Learning Stress,” “Online class,” and “Overuse of mobile phone” were formulated. See Table 2 for the classification matrix). For quantitative data, the analysis involved descriptive statistics, with data expressed as the mean ± SD or frequency. Age and duration of disorder data were used for comparisons between the groups using the *T*-test, and Cohen's d was used to describe the effect size of the differences. The chi-square test was used to determine the differences in psychosocial factors related to NSSI between the pre-pandemic group and the during-pandemic group. Binary logistic regression models were performed using the forward stepwise method to explore the psychosocial factors associated with NSSI among discharged adolescents, and odds ratio (OR) and 95% confidence intervals (CIs) were obtained to show the associations between these factors and the outcomes. The sample of discharged adolescents that did not engage in NSSI after the outbreak of the pandemic was small, and the number of factors was relatively large, resulting in the restriction of using logistic regression models, so Spearman's correlation was used to examine the association between NSSI behaviours and psychosocial factors in the different groups. A *p* < 0.05 was considered statistically significant. The statistical software used for all analyses was SPSS, version 20.0 (IBM-SPSS Inc., Armonk, NY, USA).

**Table 1:** The demographic characteristics and psychosocial factors related to NSSI among adolescents admitted to our hospital.

**Variable and assignments**	**NSSI group** **(Total ***n*** = 420)** **(***N*** %)**	**Without NSSI group ** **(Total ***n*** = 189)** **(***N*** %)**	***OR*** **(95% CI)**	***p-*** **value**
**Gender**			0.075 (0.029, 0.190)	<0.001
Female (1)	402 (95.7%)	130 (68.8%)		
Male (2)	18 (4.3%)	59 (31.2%)		
**Average age** (years ± SD)	15.33 ± 1.74	15.37 ± 1.75	1.215 (1.022, 1.444)	0.023
**Average duration of disease** (months ± SD)	15.42 ± 8.24	14.83 ± 7.08	1.021 (0.978, 1.065)	0.364
**Frequency of treatment episodes** (times ± SD)	0.34 ± 0.67	0.39 ± 0.75	0.882 (0.587, 1.326)	0.547
**Single parent**			7.751 (3.951, 15.205)	<0.001
Yes (1)	226 (53.8%)	33 (17.5%)		
No (0)	194 (46.2%)	156 (82.5%)		
**Only child**			0.713 (0.396, 1.287)	0.278
Yes (1)	124 (29.5%)	77 (40.7%)		
No (0)	296 (70.5%)	112 (59.3%)		
**Experienced trauma**			2.214 (1.043, 4.700)	0.041
Yes (1)	75 (17.9%)	27 (14.3%)		
No (0)	345 (82.1%)	162 (85.7%)		
**Social isolation from peers**			8.313 (4.134, 16.716)	<0.001
Yes (1)	239 (56.5%)	24 (12.7%)		
No (0)	181 (43.1%)	165 (87.3%)		
**Hurt by information on social media**			1.082 (0.434, 2.696)	0.870
Yes (1)	115 (27.4%)	13 (6.9%)		
No (0)	305 (72.6%)	176 (93.1%)		
**Body-focused bullying**			3.116 (1.311, 7.405)	0.011
Yes (1)	109 (26.0%)	12 (6.3%)		
No (0)	311 (74.0%)	177 (93.7%)		
**Parents'**			0.812 (0.438, 1.504)	0.489
misunderstanding^*^			
Yes (1)	252 (60.0%)	76 (40.2%)		
No (0)	168 (40.0%)	113 (59.8%)		
**Online class**			1.494 (0.304, 7.333)	0.601
Yes (1)	181 (43.1%)	6 (3.2%)		
No (0)	239 (56.9%)	183 (96.8%)		
**Learning stress**			1.702 (0.793, 3.656)	0.191
Yes (1)	228 (54.3%)	26 (13.8%)		
No (0)	192 (45.7%)	163 (86.2%)		
**Mobile phone overused**			4.199 (1.343, 13.122)	0.012
Yes (1)	201 (47.9%)	9 (4.8%)		
No (0)	219 (52.1%)	180 (95.2%)		
**Attempted suicide**			9.276 (4.580, 18.783)	<0.001
Yes (1)	289 (68.8%)	23 (12.2%)		
No (0)	131 (31.2%)	166 (87.8%)		
**Before/during pandemic**			5.421 (1.693, 17.352)	0.005
During (1)	266 (63.3%)	14 (7.4%)		
Before (0)	154 (36.7%)	175 (92.6%)		
**Constant**	–	–	0.063	0.062

* *The item of “Parents' mis-understanding” stands for the psychosocial factor “Parent–child relationships”*.

**Table 2:** The unconstrained categorization matrix of psychosocial factors of NSSI.

**What are the psychological factors related to NSSI?**	
**Experienced trauma**	Earthquake Traffic accident Sexual assault ……
**Body-focused bullying**	Be slapped in the face Be besieged Be pulled clothes down ……
**Social isolation from peers**	Neglected by peers No one to play with Being kept distance ……
**Hurt by information on social media**	Be attacked by words on the internet ……
**Parent–child relationships**	Misunderstood by parents
**New categories to be formulated by inductive content analysis**	……

## Results

### The Demographic and Clinical Characteristics of All Included Adolescents

Among the 609 adolescents, 43.7% (266/609) came from Chengdu, 44.5% (271/609) came from other cities of Sichuan Province, and the rest came from other provinces. The top four pre-valences of psychiatric disorders among them were “behavioural and emotional disorders that usually occur in childhood and adolescence” (33.5%), “depressive disorders” (24.6%), “anxiety disorders” (13.1%), and “bipolar disorder” (10.0%). The remaining diagnoses included “post-traumatic stress disorder” (4.8%), “personality disorders” (4.3%), “obsessive-compulsive disorder” (3.1%), “conversion disorder” (2.6%), and “adjustment disorder” (0.5%). When grouped according to their behaviours, 420 subjects had engaged in NSSI behaviours, while 189 had not; we classified the former into the “NSSI group,” and the latter into the “without NSSI group.” In the “NSSI group,” the top three disorders were “behavioural and emotional disorders that usually occur in childhood and adolescence” (48.6%), “depressive disorders” (22.1%), and “bipolar disorder” (8.8%), while the top three disorders in the “without NSSI group” were “anxiety disorders” (33.3%), “depressive disorders” (30.2%), and “bipolar disorder” (12.7%). The demographic details of these two groups are shown in Table 1. In the NSSI group, the disorder duration ranged from 5 to 72 months, and the age ranged from 10 to 18 years, while the corresponding ranges in the other group were 6–36 months and 12 to 18 years. In the NSSI group, the proportion of patients who got their first treatment for their psychiatric disorders in our ward was 76.2% (320/420), while the proportion in the “without NSSI group” was 73.5% (139/189).

### The Distribution of Adolescents That Engaged in NSSI for Each Year

**Figure 1:**
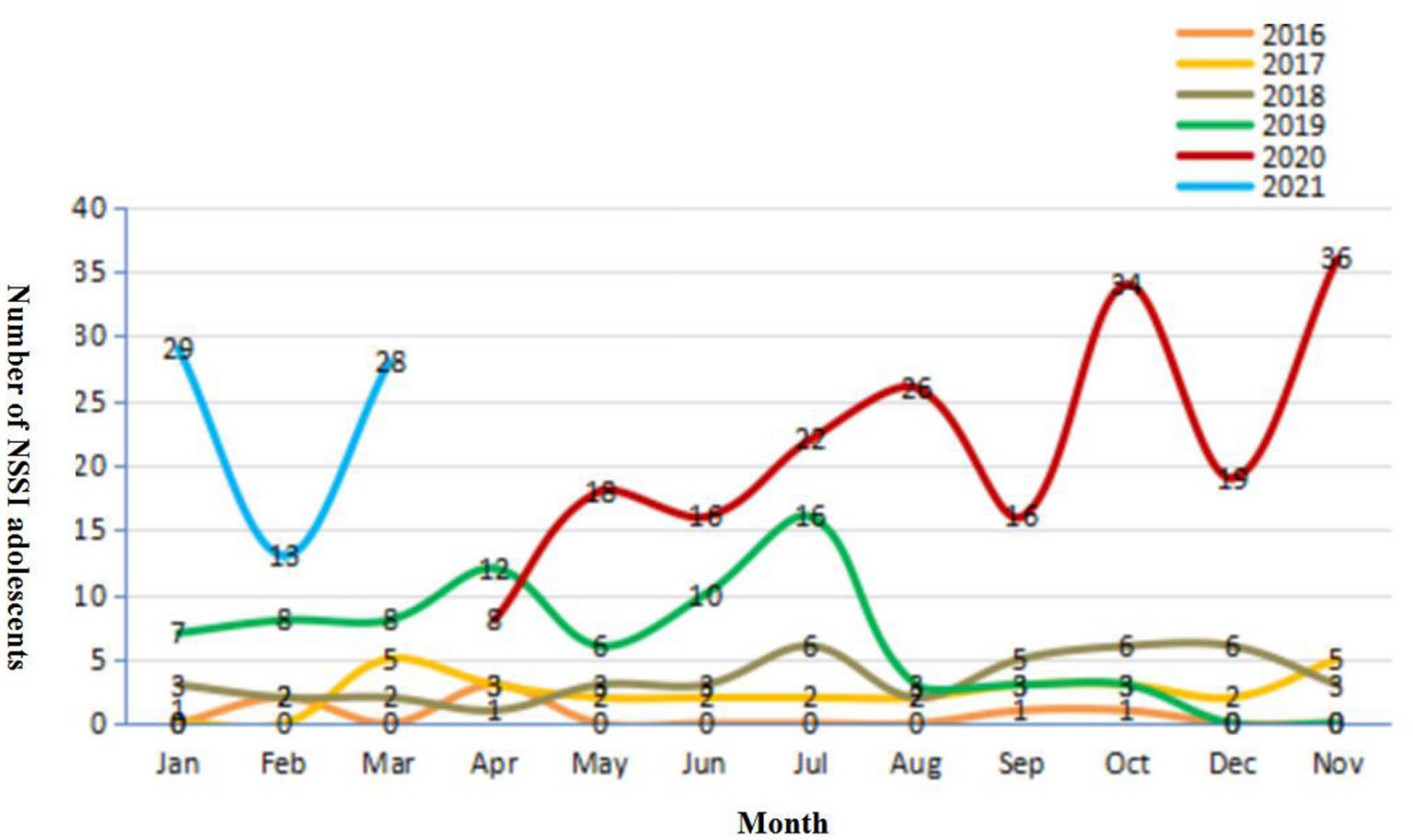
The number of NSSI adolescents in each month from 2016 to 2021. From February 2020 to March 2020, our ward was shut down due to the pandemic, so the line for these 2 months was broken.

The percentage of adolescents who engaged in NSSI in 2016 was only 29.2% (7/24), reaching 34.5% (29/55) in 2017, 45.7% (42/92) in 2018, 61.3% (76/124) in 2019, 92.5% (196/212) in 2020, and 95.9% (70/73) in 2021 (each adolescent was only included in one of these years). We found that since 2020, the proportion of adolescents who engaged in NSSI has increased dramatically. Figure 1 displays the number of adolescents who engage in NSSI in each month from 2016 to 2021. We can see that after the central provinces and cities gradually resumed work (due to the outbreak of COVID-19, many cities in China stopped production), that is, since May 2020, the number of hospitalized adolescents who engage in NSSI has increased further. We can see that the number in February 2021 was relatively small. Because it was the traditional Spring Festival, most Chinese adolescents would not want to be hospitalized during this period.

### The Related Psychosocial Factors of NSSI in All Discharged Adolescents

We took NSSI behaviours as the dependent variable; 0 indicates no NSSI behaviours, and one indicates having NSSI behaviours. All demographic characteristics, the frequency of treatment episodes, the main eight psychosocial factors abstracted from their medical records, whether the adolescents had attempted suicide previously, and the item “Before/During the pandemic” were considered independent variables, and their assignments are shown in Table 1. The association of the psychosocial factors with NSSI is presented in Table 1. In the binary logistic regression model, female sex (OR = 0.073, 95% CI: 0.028–0.186), older age (OR = 1.234, 95% CI: 1.030–1.478), having a single parent (OR = 7.865, 95% CI: 3.997–15.476), having experienced trauma (OR = 2.192, 95% CI: 1.032–4.654), having experienced social isolation from peers (OR = 8.139, 95% CI: 4.037–16.408), having experienced body-focused bullying (OR = 3.078, 95% CI: 1.295–7.318), overusing a mobile phone (OR = 4.354, 95% CI: 1.380–13.738), having attempted suicide (OR = 9.120, 95% CI: 4.492–18.512), and during the pandemic (OR = 5.399, 95% CI: 1.679–17.357) were the factors that were significantly associated with NSSI in discharged adolescents. The Omnibus tests of model coefficients showed that the model was significant (*χ*^2^ = 448.012, df = 16, *p* < 0.001), and the Nagelkerke *R* square was 0.733, which meant that 73.3% of the variance could be explained by this model.

### The Differences in the Demographic Characteristics and Incidence Rate of Psychosocial Factors Between the Different Groups

**Figure 2:**
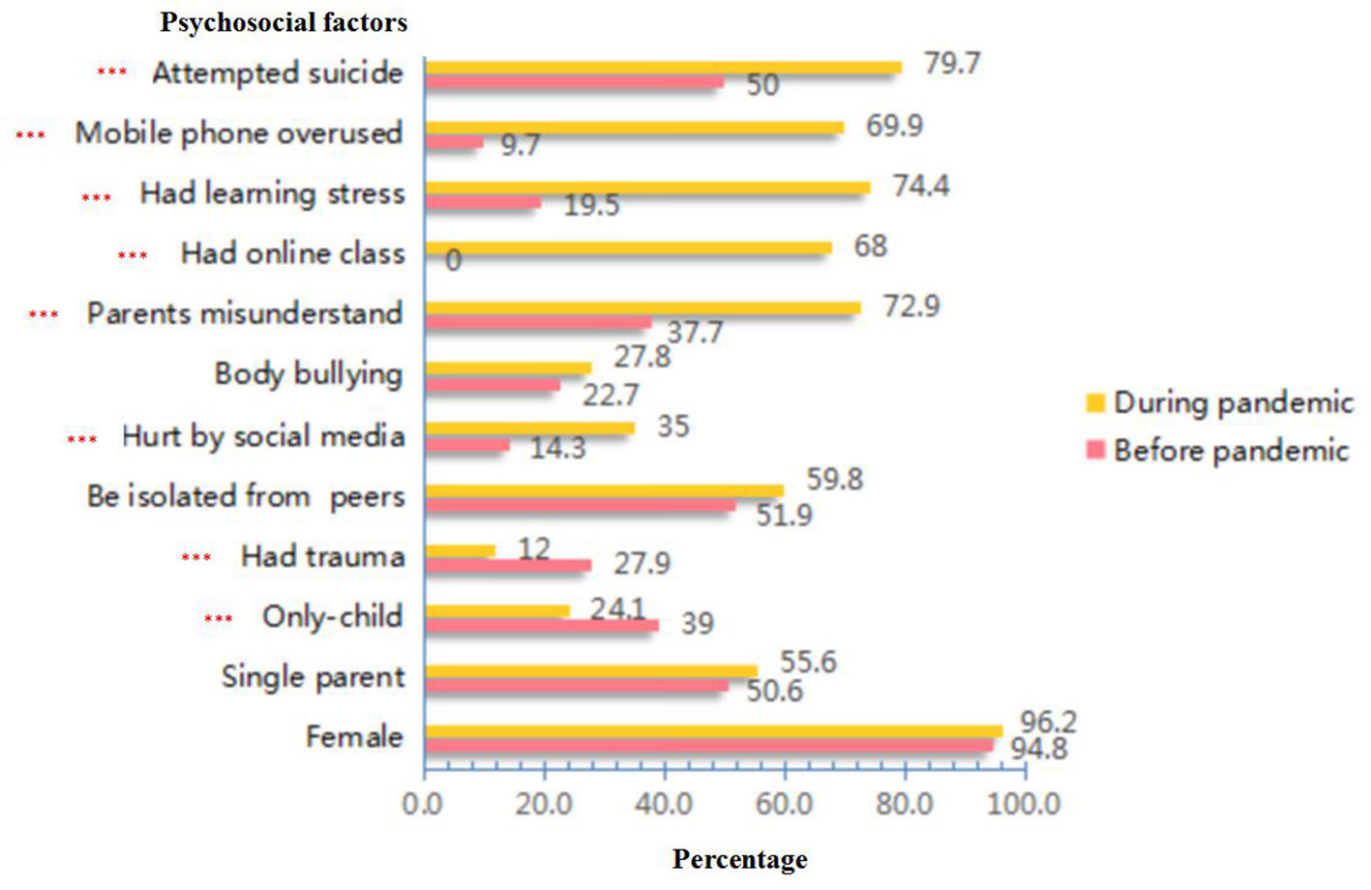
The comparison of incidence rates of related psychosocial factors among NSSI adolescents in different periods. ^***^stands for *P* < 0.001.

Through the results of the logistic regression model, we demonstrated that adolescents admitted to our hospital during the pandemic were more likely to engage in NSSI. To explore the reasons, we divided all subjects into two groups. One group consisted of subjects admitted to our hospital before the pandemic (*n* = 329), and another consisted of those admitted during the pandemic (*n* = 280). The average age of all the adolescents in the pre-pandemic group (15.58 ± 1.68 years) was significantly higher than that in the during-pandemic group (15.06 ± 1.78 years), and their disorder duration (14.59 ± 7.32 months) was significantly shorter than that in the during-pandemic group (16.00 ± 8.47 months) (*t* = 3.674, −2.215; *p* < 0.001, *p* = 0.027; the Cohen's d was 0.300 and 0.178, respectively). We also found that the average age of adolescents with NSSI behaviours in the pre-pandemic group (15.83 ± 1.60 years) was significantly higher than that in the during-pandemic group (15.06 ± 1.80 years), and their disorder duration (14.25 ± 7.42 months) was significantly shorter than that in the during-pandemic group (16.10 ± 8.61 months) (*t* = 4.409, −2.227; *p* < 0.001, *p* = 0.026, the Cohen's d was 0.452 and 0.230, respectively). We used the chi-square test to compare the incidence rate of all the psychosocial factors between the adolescents who engaged in NSSI in the pre-pandemic group and those in the during-pandemic group and discovered that after the outbreak of the pandemic, fewer adolescents who engaged in NSSI were only children (*χ*^2^ = 10.408, df = 1, *p* = 0.001), fewer adolescents had experienced trauma (*χ*^2^ = 16.793, df = 1, *p* < 0.001), more adolescents were hurt by the information on social media (*χ*^2^ = 20.971, df = 1, *p* < 0.001), more adolescents thought their parents did not understand them (*χ*^2^ = 50.554, df = 1, *p* < 0.001), more adolescents took online classes (*χ*^2^ = 184.149, df = 1, *p* < 0.001), more adolescents thought their learning was stressful (*χ*^2^ = 118.697, df = 1, *p* < 0.001), more adolescents' parents thought their children overused mobile phones (*χ*^2^ = 141.573, df = 1, *p* < 0.001), and more adolescents had previously attempted suicide (*χ*^2^ = 40.084, df = 1, *p* < 0.001) (see Figure 2 for details).

### The Correlation Between NSSI Behaviours and Related Psychosocial Factors in the Different Groups

We used Spearman's correlation to analyse the relationship between NSSI behaviours and all psychosocial factors in the pre-pandemic group and during-pandemic group. The results are shown in Table 3. The influencing factors in the two groups were different (see the bold font in Table 3), which illustrated that the pandemic had changed the potential psychosocial factors for NSSI behaviours.

**Table 3:** The correlation between NSSI and psychosocial factors in different groups.

**Factors**	**NSSI status**	
	**Pre-pandemic** **(***n*** = 329)**	**During-pandemic** **(***n*** = 280)**
**Age**	0.140^*^	−0.044
Gender	−0.317^**^	−0.422^**^
Duration of disorder	−0.043	0.050
Frequency of treatment episodes	−0.026	−0.009
Single parent	0.349^**^	0.181^**^
**Only child**	−0.005	−0.165^**^
**Experienced trauma**	0.160^**^	0.033
Social isolation from peers	0.418^**^	0.232^**^
Hurt by information on social media	0.122^*^	0.128^*^
**Body-focused bullying**	0.237^**^	0.102
**Parents' misunderstanding**	0.005	0.299^**^
Online class	–	0.117
**Learning stress**	0.069	0.323^**^
**Mobile phone overused**	0.088	0.323^**^
Attempted suicide	0.422^**^	0.301^**^

* *p < 0.05*;

** *p < 0.01. The coding rules for the binary variables: for the NSSI status, 0 stands for without NSSI, and 1 for engaged in NSSI. For other binary variables, such as gender and being a single parent, the specific assignments were the same as listed in Table 1*.

## Discussion

This is an important study to understand the changing trend of NSSI behaviours and the related psychosocial factors among adolescents in psychiatric hospitals during the pandemic. We found that after the outbreak of COVID-19, the number of adolescents with NSSI behaviours admitted to the hospital increased, and their average age was younger, while their duration of disorder was longer, which means that the seriousness and difficulty of the problem increased.

In Figure 1, we can tell that the incidence of patients with NSSI behaviours in 2019 appeared to show a similar trajectory to 2020 until the month of August (except for the period of the Spring Festival and the shutdown of our ward in 2020), which shows an obvious increase compared to previous years. It could be implied that, prior to the current pandemic, the prevalence of NSSI behaviours among adolescents has increased, just as the findings of Gillies et al. identified a significant increase in the estimated lifetime prevalence of self-harm over time from 1990 to 2015 (29). The reason might be the growing pressure from social networks and the propagation effect of the media (11, 30). However, we also found that after the outbreak of the pandemic, the number of adolescents with NSSI behaviours continued to rise, which might indicate the persistent negative impact of the pandemic.

When analysing the influencing factors of NSSI in all adolescents admitted to our hospital, we found that females were more prone to engage in NSSI behaviours, which is consistent with findings from previous studies on NSSI behaviours in adolescents (31, 32). We also found that parents' divorce, having experienced trauma, having experienced social isolation from peers, and having experienced body-focused bullying were all associated with adolescents' NSSI behaviours. These psychosocial factors screened out in our study have been reported by many similar studies (33–35). Another important influencing factor in our study was the item of attempted suicide, which is further supported by the study by Wolff et al. that stated that more than 70% of hospitalized adolescents with NSSI behaviours had ever attempted suicide or committed suicide (36). This finding is also similar to the report of Fox of a significant association between NSSI behaviours and prior suicidal thoughts/behaviours (37). In contrast to other studies, we found that the overuse of mobile phones deemed by parents was significantly associated with adolescents' NSSI behaviours, and the outbreak of the pandemic did become another influencing factor of NSSI behaviours. The reasons are discussed in the next paragraph.

When analysing the results, we cannot help but to ask why the pandemic aggravated the appearance of NSSI behaviours. Studies have found that the pandemic does have a particular impact on mental health, leading people to be more prone to be anxious and depressed (38). However, apart from the psychological pressure brought by the pandemic, what factors lead these adolescents to hurting themselves repeatedly and being sent to the hospital? We might determine the answer by comparing the incidence of psychosocial factors between different groups. After the outbreak of the pandemic, great changes have taken place in the structure of adolescents' lives. First, due to the delay of returning to work because of the COVID-19 pandemic, parents and adolescents are with each other day and night, so parents may have a greater awareness of their children's self-injury behaviours, similar to the results that Hasking et al. mentioned in their study (19). This could impel parents to change their decision to seek professional medical help. When NSSI occurs, parents might try hard to control their children's behaviours. In turn, adolescents might think this control is an intrusion, which might then lead to an increased risk for NSSI (25). Second, the time spent together inevitably increased communication. As parents and adolescents always insist their own opinions are correct instead of putting themselves in each other's shoes, the conflicts brought by misunderstanding might intensify. When conflicts rose to a certain level that adolescents could not endure, they started to hurt themselves habitually to alleviate the pain caused by the contradiction (39). Therefore, 72.9% of adolescents with NSSI behaviours in the during-pandemic group thought their parents did not understand them, and this misunderstanding was also proven to be significantly correlated with the appearance of NSSI behaviours. The latest study discovered that the COVID-19 pandemic is associated with impaired distress tolerance of family members and may reduce satisfaction among parents and their children (19). Third, due to the pandemic, most schools had to postpone their opening dates. To avoid cancelling the courses, the schools carried out distant learning as a new teaching mode, which might bring long-term consequences to students (40), which is similar to the report by Sindiani et al. that found that online classes could not provide a calm environment for students and might bring lower academic achievement (41). The limited class interaction and inefficient timetable might reduce students' satisfaction (42). Many adolescents thought the teaching mode for online courses made it difficult to understand the class materials, which may further increase their anxiety about learning. This could be proven by the high rate of 74.4% for learning stress in the during-pandemic group, and this factor was also found to be correlated with NSSI behaviours during the pandemic. Finally, the increased time of using electronics during the pandemic may also increase parents' anxiety transmitted to their children, which could be echoed by the opinions of these parents. We found that 69.9% of the parents of adolescents with NSSI behaviours in the during-pandemic group complained that their children spent too long playing on mobile phones, which successfully included this factor into the sequence of NSSI influencing factors. Chaturvedi et al. discovered a significant increase in the use of social media as a medium for stress relief in adolescents after the outbreak of the pandemic (42). For adolescents with emotional problems, the virtual world might be safer and could help them calm down, which could be verified by the finding that mobile phone use was positively correlated with anxiety (43). Furthermore, mobile phone use was found to be negatively correlated with a good parent–child relationship (43). Adolescents who use mobile phones excessively might have a bad relationship with their parents, and a tense parent relationship has been proven to be a risk factor for NSSI (23). In addition to the risk factors mentioned above, we also found that adolescents from multiple child families were more likely to engage in NSSI during the pandemic. The possible reason might be the competitive interaction between siblings. With the liberalization of the second child policy, an increasing number of Chinese families have at least two children. The proportion of only child adolescents with NSSI behaviours in the during-pandemic group was only 24.1%, which was significantly lower than that in the pre-pandemic group. Most of them had to suffer the role of taking care of their younger siblings, and according to a related study, it was found that these adolescents might consider caring for young siblings to be stressful, and had the feeling of deprived love from their parents (44, 45), which might further lead to NSSI behaviours.

Regarding the composition change of related psychosocial factors of NSSI behaviours, we found that after the outbreak of the pandemic, experiencing trauma and suffering body-focused bullying played decreased roles in the appearance of NSSI behaviours, while the family constitution, parents' misunderstanding, mobile phone overuse, and stressful learning have become important factors. It is imperative to plan tailored strategies to prevent NSSI behaviours from the factors listed above. First, schools can provide relevant education for adolescents on how to cope with stress, from passive coping with NSSI behaviours to active mental health education and guidance, thus providing a suitable environment for their healthy growth. As recommended by Singh et al., teachers can conduct academic and non-academic sessions in their classes and play a role in the promotion of mental health among the students (16). Second, it is also vital for parents to learn how to understand and contain their children's emotions beyond paying attention to academic achievements, because parents' rejection and neglect in family rearing patterns are related to teenagers' self-injury behaviours (46). If parents can master a reasonable way to manage emotions and guide their children in times when their children have difficulties, they will avoid intensifying conflicts. As Paul once pointed out, providing health education and skills training for the families of adolescents who engage in NSSI would be very beneficial (47). Regarding the problem of mobile phone overuse, if the parents stop their children's use in a simple and disrespectful way, it might further deteriorate their dysfunctional emotions. Parents are recommended to learn how to negotiate with adolescents to limit their time in an understanding and respectful manner. In addition, if schools can contact psychotherapists or psychiatrists regularly to assist in developing emotional processing skills such as dialectical behaviour therapy (DBT) for teachers and parents, it would greatly benefit children with emotional problems (48). The research conducted by Singh et al. also suggested that it is vital to ameliorate adolescents' access to mental health support services and to establish collaborative networks with psychiatrists and psychologists (16).

## Limitations

Our study should be interpreted in light of the following limitations. First, all data came from the medical records in the electronic system, and it was impossible to acquire the objective risk factors in terms of scale scores. Second, the subjects in our study came from the same ward, and their representativeness would lead to the limitation of extending the conclusions to a wider population. There is a pressing need to plan a strong and evidenced-based follow-up study to understand the changing trend of NSSI behaviours among adolescents. Third, if we can conduct an in-depth analysis of the patterns and times of adolescents' self-injury behaviours, our results will be richer.

## Conclusions

In this study, we not only discovered a dramatic increase in adolescents with NSSI behaviours during the pandemic but also examined the structural changes in NSSI risk factors over time. The results showed that family constitution, relationship with their parents, mobile phone overuse, and stressful learning had significant association with NSSI behaviours during the pandemic, which were distinguished from those before the pandemic (49).

## Data Availability Statement

The raw data supporting the conclusions of this article will be made available by the authors, without undue reservation.

## Ethics Statement

The studies involving human participants were reviewed and approved by the Fourth People's Hospital of Chengdu. Written informed consent to participate in this study was provided by the participants' legal guardian/next of kin.

## Author Contributions

ND: conception and design and drafting of the manuscript. ND, YO, and YX: conduction and statistical analysis. YL: administrative, technical, or material support. YO and YX: critical revision of the manuscript for important intellectual content. All authors read and approved the final paper.

## Funding

This research was funded by the Chengdu hygiene and health Committee. Award Number: 2019025.

## Conflict of Interest

The authors declare that the research was conducted in the absence of any commercial or financial relationships that could be construed as a potential conflict of interest.

## Publisher's Note

All claims expressed in this article are solely those of the authors and do not necessarily represent those of their affiliated organizations, or those of the publisher, the editors and the reviewers. Any product that may be evaluated in this article, or claim that may be made by its manufacturer, is not guaranteed or endorsed by the publisher.
